# Hypertrophic cardiomyopathy with little hypertrophy and severe arrhythmia

**DOI:** 10.21542/gcsp.2018.26

**Published:** 2018-08-12

**Authors:** Tomas Ripoll-Vera, Jorge Alvarez-Rubio

**Affiliations:** CIBER Fisiopatología Obesidad y Nutrición (CIBERobn), Instituto de Salud Carlos III, Madrid, Spain; Balearic Islands Health Research Institute (iDisBA),*Inherited Cardiovascular Disease Unit, Cardiology Department, Hospital Universitari Son Espases, Edifici S, Carretera de Valldemossa 79*, 07120 Palma, Illes Balears Spain; Son Llatzer University Hospital, Carretera de Manacor km.4, 07198 Palma, Illes Balears, Spain

## Introduction

Hypertrophic cardiomyopathy (HCM) is an inherited autosomal-dominant disease with a heterogeneous clinical presentation and natural history^1^, and is a frequent cause of sudden cardiac death (SCD) in young people^2–4^. It is associated with mutations in genes coding for sarcomere proteins^5–7^. In the literature, debate surrounds the genotype-phenotype correlation of individual mutations^7,8^ concerning establishing a prognosis according to the mutation present, which could help stratify the disease and allow appropriate genetic counselling to families.

In an adult, HCM is defined by a wall thickness ≥15 mm in one or more left ventricular (LV) myocardial segments—as measured by any imaging technique (echocardiography, cardiac magnetic resonance imaging (CMR) or computed tomography)—that is not explained solely by loading conditions^9^, but there are cases of HCM with thicknesses of less than 15 mm and even cases without hypertrophy, all documented by the presence of disarray in the histopathological study of the heart, given that they are also cases with high arrhythmic risk and therefore SCD.

In the latest guidelines of the European Society of Cardiology^9^ this circumstance is scarcely mentioned. Only the following text refers to it specifically: “*Genetic and non-genetic disorders can present with lesser degrees of wall thickening (13–14 mm)”.* It is also mentioned in the section on Diagnostic Challenges: *“Common diagnostic challenges include the following:*

• Presentation in the late phase of the disease with a dilated and/or hypokinetic left ventricle and LV wall thinning
• Physiological hypertrophy caused by intense athletic training
• Patients with co-existent pathologies
• Isolated basal septal hypertrophy in elderly people”

In children, the diagnosis of HCM requires an LV wall thickness more than two standard deviations greater than the predicted mean (z-score >2, where a z-score is defined as the number of standard deviations from the population mean).

Therefore, cases with very little hypertrophy and a lot of fibrosis or disarray do not appear in the clinical guidelines, and there is no reference to their management and prognosis.

**Table 1:** Studies published on survival in *TNNT2* gene mutations.

Studies	Number of families	Number of patients	Number of cardiac deaths	Number of sudden deaths	Mutation
Watkins^9^ 1995	11	112	50	39	Ile79Asn Arg92Gln Phe110Ile ΔGlu160 Glu163Lys Glu244Asp Intron 15 G>A Arg278Cys
Nakajima-Tanaguchi^22^ 1997	1	4	2	2	Ala104Val
Moolman^11^ 1997	2	22	7	7	Arg92Trp
Anan^17^ 1998	6	18	2	2	Phe110Ile
Torriceli^18^ 2003	5	10	0	0	Phe110Ile Arg130Cys ΔGlu160 Arg92Gln Arg278Cys
Pasquale^23^ 2012	20	92	¿?	7	Arg278Cys Arg92Leu Arg92Trp ΔGlu163 IVS15+1G>A Ala104Val, Arg278His Arg92Gln Arg94Leu Glu163Lys Glu83Lys Ile79Asn
Ripoll-Vera 2016	21	54	11	6	Arg92Gln Arg92Trp Arg286His Arg278Cys Arg94His Ile221Thr

**Figure 1.**
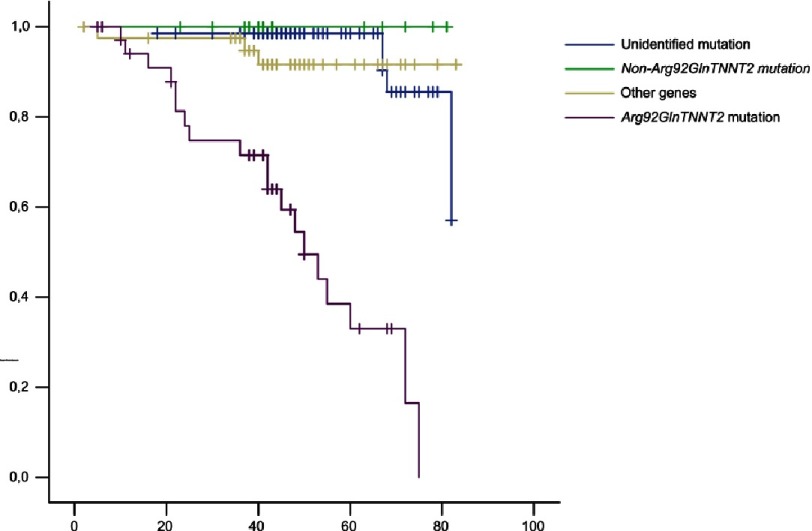
Free survival of sudden cardiac death, including patients with recovered SCD and patients with appropriate ICD therapies, depending on the genetic result.

**Figure 2.**
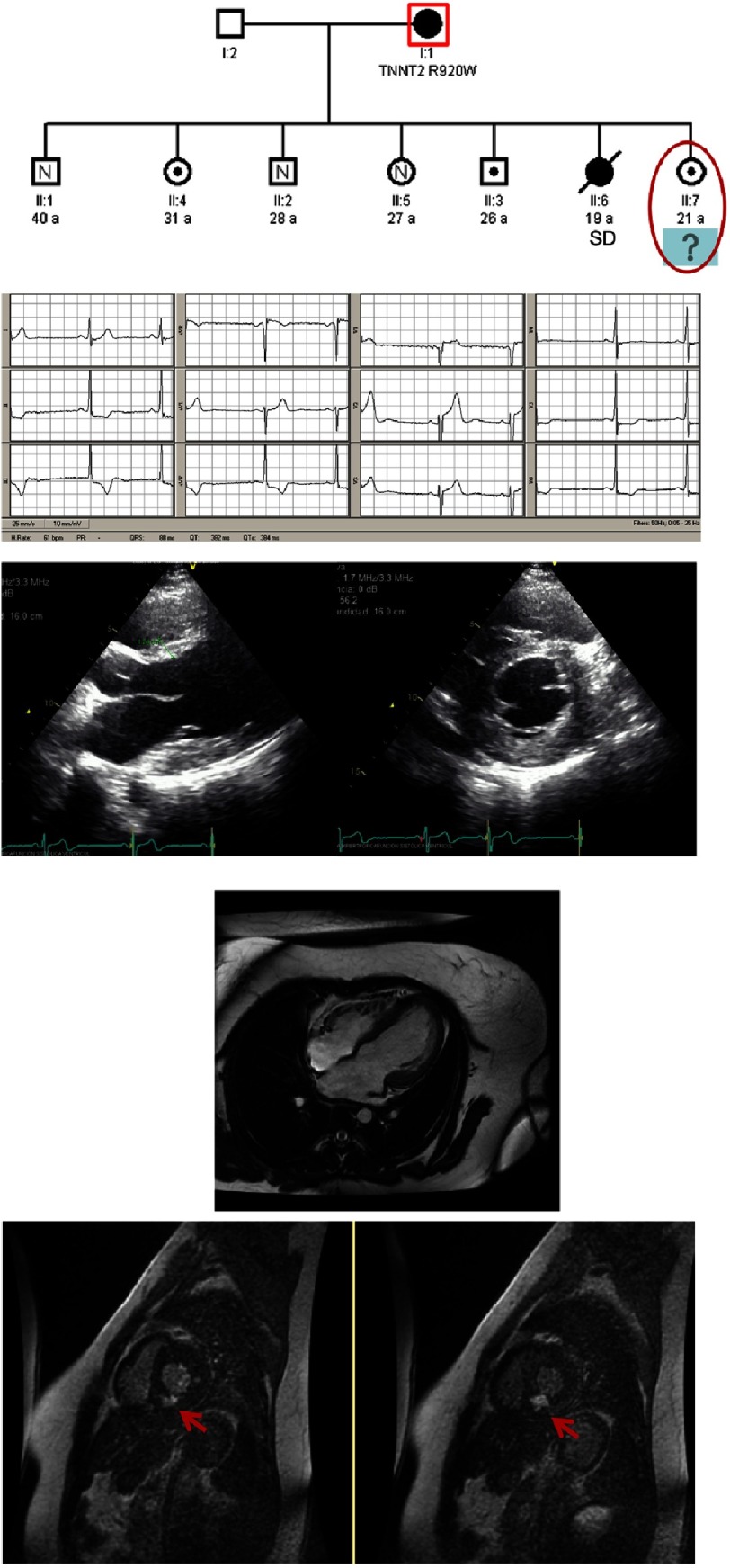
Clinical case of a family: the proband is a 19 years old man with a SCD. His mother had an HCM and is carrier of a mutation in *TNNT* gene (*Arg92Trp*). The pedigree shows that there are also 2 sisters and 1 brother carrying the same mutation. Images from the youngest sister are shown: a pathological electrocardiogram with ST elevation in right precordial leads and negatives T waves in lateral and inferior wall, and a TTE and CMR showing a normal LV wall thickness, except for the posterior wall (mild hypertrophy) and a severe amount of fibrosis in this localization (red arrows).

Mutations in the troponin T gene (*TNNT2*) were described years ago in several publications with few families, and researchers postulated a high prevalence of SCD in young carriers^5,6,10,11^, who, in addition, had a phenotype of mild left ventricular hypertrophy^6,12^.

The first cases were described in 1990 by McKenna et al.^13^, and the first mutation in *TNNT2* gene in 1999 by the same group^14^. Mutations in *TNNT2* represent around 5% of cases of HCM. They have been described as associated with moderate or mild hypertrophy with a poor prognosis due to a high risk of SCD, even in the absence of hypertrophy, with early expression in adolescence, based mainly on the study by Watkins et al.^10^, in which 11 families with 8 different mutations were described.

More recently, two series with a greater number of families have been published^15,16^ (Pasquale et al., 2012, 20 families and 12 mutations, and Ripoll-Vera et al., 2016, 21 families and 6 different mutations) (Table 1). In these last series it was found that in the *TNNT2* gene HCM up to 19% the ECG is normal and in 23% the transthoracic echocardiography (TTE) does not show hypertrophy. On the contrary, up to 24–48% non-sustained ventricular tachycardia is documented. However, we must differentiate which mutations we are talking about. Not all mutations in the *TNNT2* gene have a poor prognosis (Figure 1).

The *Arg92Gln* mutation is the most studied, it can manifest as HCM with little hypertrophy, but also as dilated cardiomyopathy, especially at older ages. The high burden of SCD is a constant in these families. Of 15 cases documented in 10 families, SCD was the first manifestation of the disease in all, at an average age of 21 years (range 11–42) and with an average myocardial thickness of 14.6 ± 5.2 mm. Up to 40% of patients required the implantation of an implantable cardiac defibrillator (ICD), either for primary or secondary prevention. The *Arg94Leu* mutation, like *Arg94Cys* and *Arg94His*, that affect the same residue, also behave with a certain malignancy^17^. Microscopic evaluation in some studies in carriers of these *TNNT2* mutations indicate that cause less hypertrophy and fibrosis than other sarcomeric mutations, but more disarray. This may be the substrate that explains the high arrhythmic risk^18^.

Conversely, other mutations such as *Arg278Cys* or *Arg286His*, also in the *TNNT2* gene, have a much more benign course^16^.

The diagnosis of these patients is therefore complex, and in some cases an ECG and a TTE will not be enough. CMR has shown its usefulness against TTE in the detection of mild hypertrophies or in the evaluation of worst viewed segments by TTE^19^. Even in those cases with little or no hypertrophy, the presence of late gadolinium enhancement is a risk marker for SCD in these patients. It has been shown that in these cases, where there is an absence of the classic risk factors of SCD in HCM, the presence of more than 15–20% of fibrosis is a strong and independent marker of outcomes. Even Maron et al.^20^ proposed an algorithm in which it is emphasized that a HCM with extensive fibrosis and absence of conventional risk factors of SCD, an ICD should be implanted as primary prevention.

Recently, new techniques have been incorporated in CMR such as T1 mapping and quantification of extracellular volume (ECV), which seem to have a manifest utility as an early marker of the disease, even when fibrosis has not yet appeared (Figure 2).

## Conclusions

a) The current definition of HCM does not cover all possible phenotypes, because HCM with little or even “no hypertrophy” exists and can have a very bad prognosis, with SCD at young ages, being very frequently the first manifestation of the disease.
b) In histopathology, a lot of disarray is noticeable in this particular phenotype.
c) It is especially related to some mutations in *TNNT2*. The clinical and prognostic profiles depended greatly on the specific mutation.
d) A common factor is usually the presence of family history of SCD.
e) To stratify the risk of the carriers, we can not trust only the ESC risk score or the classic American criteria.
f) CMR is essential to identify hypertrophy in segments that are more “hidden” to TTE and to evaluate the presence of fibrosis, which is a very important risk marker in these patients and can help us define which patients need an ICD.
g) Investigation of the genotype-phenotype correlation in HCM remains a challenge.
h) Overall, these findings have important implications for the clinical and genetic study of families with cardiomyopathy, above all the findings of some *TNNT2* mutations, which, given its demonstrated malignancy, should cause a change in the management of individuals in SCD prevention.
